# Recurrent Acute Pancreatitis Probably Induced by Rosuvastatin Therapy: A Case Report

**DOI:** 10.1155/2012/973279

**Published:** 2012-03-26

**Authors:** JayaKrishna Chintanaboina, Deepa Gopavaram

**Affiliations:** Wright Center for Graduate Medical Education, 501 Madison Ave, Scranton, PA 18510, USA

## Abstract

*Context*. Approximately 1.4–2% of all cases of acute pancreatitis are drug related in general population. The literature on statin-induced pancreatitis consists primarily of anecdotal case reports. We report a case of possible rosuvastatin-induced pancreatitis. *Case Report*. A 67-year-old female presented with progressively worsening abdominal pain and vomiting for 7 days. Home medications included rosuvastatin and clonidine. CT scan of abdomen, with intravenous contrast, showed findings consistent with acute pancreatitis. She responded to conservative management. Rosuvastatin was resumed at the time of discharge from the hospital, and she presented two months later with recurrence of acute pancreatitis. Further workup ruled out all likely causes of acute pancreatitis. Rosuvastatin was stopped completely when she was discharged the second time, and she did not have any further episodes of acute pancreatitis. She was completely asymptomatic throughout the 18-month follow-up period. *Conclusion*. This paper reinforces the possible association of rosuvastatin, a novel statin, with acute pancreatitis, even though the exact underlying mechanism of statin-induced pancreatitis remains unknown.

## 1. Introduction

Several causes of acute pancreatitis (AP) have been established, but the most common are gallstones (30–60%) and alcohol abuse (15–30%) [1]. Other common causes of AP include hypertriglyceridemia, hyperparathyroidism, endoscopic retrograde cholangiopancreatography (ERCP), trauma, pancreatic tumors, and surgery (intraabdominal and nonabdominal) [2]. Drugs are a relatively uncommon cause of AP and account for 1.4–2% of the cases in the general population [3]. Although anecdotal case reports have claimed that statins cause AP, the exact incidence and mechanism of this occurrence is unknown. We report a case of 67-year-old female who developed AP during treatment with rosuvastatin; that was resolved upon discontinuation of the drug, and it recurred after readministration of rosuvastatin while other likely causes of AP were ruled out.

## 2. Case Report

A 67-year-old female with a past medical history of hypertension and dyslipidemia presented with nausea, vomiting, and epigastric pain for 7 days. She admitted to a history of multiple drug intolerances. A few days prior to the presentation, the patient was started on oral rosuvastatin 10 mg daily for dyslipidemia at the discretion of the primary care physician. She denied alcohol abuse or a family history of gastrointestinal disease. Upon presentation to the emergency room, vital signs were stable and physical examination was remarkable for epigastric tenderness without guarding or rigidity and normal bowel sounds. The rectal examination was unremarkable, and the stool occult blood was negative.

The laboratory data upon presentation to the emergency room is shown in Table 1. The serum lipase was mildly elevated at 82 U/L. Liver enzymes, including serum bilirubin, were normal. The CT scan of abdomen, with intravenous contrast, showed mild edema of pancreatic and peripancreatic tissue that was confined to the head and body of the pancreas, which was consistent with acute pancreatitis. The abdominal ultrasound showed a normal biliary tree without choledocholithiasis. Rosuvastatin was held at the time of admission. She was treated conservatively with bowel rest, intravenous fluids, and parenteral pain management. She improved clinically over next 3 days, tolerated regular diet and was discharged home. Her serum lipase levels trended down to normal (i.e., 27 U/L) during the hospital course. She was restarted on rosuvastatin at the time of discharge, as there was no strong evidence of its correlation with AP at that time except for one case report. After detailed discussions regarding the risks and benefits, the patient agreed to restart rosuvastatin at the time of discharge from the hospital.

Eight weeks later, the patient presented to the emergency room with vomiting and epigastric pain for 7 days. Her vital signs were stable, and the physical examination was remarkable for epigastric tenderness and normal bowel sounds. The serum amylase and lipase were elevated at 168 U/L and 100 U/L, respectively. The laboratory data upon presentation to the emergency room is shown in Table 1. The CT scan of abdomen, with intravenous contrast, showed mild edema of the head and body of the pancreas and mild stranding of the adjacent fat, consistent with acute pancreatitis and there was no evidence of any pancreatic mass lesion. Magnetic resonance cholangiopancreatography (MRCP) did not reveal biliary sludge or microlithiasis. The serum IgG4 levels were normal, which ruled out autoimmune pancreatitis. The patient responded to bowel rest, intravenous fluids, and pain management. She clinically improved over the next three days and was able to tolerate regular diet. Serum lipase and amylase trended down to 22 U/L and 44 U/L, respectively. After reviewing case reports of statin-induced pancreatitis and ruling out other differential diagnoses, it was determined that rosuvastatin would be stopped at the time of discharge. The strength of association between rosuvastatin and AP increased during this admission as this could be considered as a drug rechallenge and all other possible diagnoses were ruled out. After treatment with rosuvastatin was terminated, the patient did not have any further episodes of AP and was completely asymptomatic during the 18-month followup period. She remained on a strict, fat-free diet and was never started on any medication for dyslipidemia, as her LDL cholesterol remained at less than 100 mg/dL during followup.

## 3. Discussion

Drug-induced pancreatitis is unusual, but the incidence may be increasing [12]. There are no distinguishing clinical features of drug-induced pancreatitis. Diagnosis of drug-induced AP is based on a high index of clinical suspicion and detailed drug history. Serum amylase and serum lipase levels are elevated at least two times the upper normal limit of lab values in AP. Serum lipase is more specific than serum amylase in the diagnosis of AP. Serum lipase levels usually decline slowly over 7–14 days after the onset of AP [13]. Serum amylase has a shorter half-life than lipase due to rapid renal clearance [14]. A CT scan of the abdomen, with intravenous contrast, is the best imaging modality to diagnose, assess severity, and identify intra-abdominal complications of AP [15]. Although CT scan of abdomen is usually indicated for identification of complications like pancreatic necrosis or abscess, it is still a valuable tool for diagnosis if the patient has clinical symptoms of acute pancreatitis and serum amylase or lipase that are not significantly elevated. Management of drug-induced pancreatitis usually includes intravenous hydration, bowel rest, nutritional support, parenteral pain management, and withdrawal of the potentially causative drug. Our patient presented 7 days after the onset of symptoms of acute pancreatitis, and serum lipase/amylase usually starts to trend down over this period of time. However, there was significant evidence of acute pancreatitis on the CT scan of the abdomen, with intravenous contrast, which is a highly specific test for the diagnosis of acute pancreatitis. Other diagnoses such as biliary disease and malignancy were ruled out by the ultrasound of the abdomen, MRCP, and serum tumor markers. The patient responded to supportive management when the rosuvastatin was stopped during both episodes of AP. Although the patient was taking clonidine upon presentation, she had not developed AP while taking this drug for several months before and after her discharge from the hospital.

Statins are recommended for primary and secondary prevention of coronary artery disease in diabetes mellitus, as they have been shown to improve mortality in several clinical trials [16]. Intensive drug therapy is recommended to keep the low-density cholesterol at a low level. Statins are the most commonly prescribed drugs for the treatment of dyslipidemia. They are usually well-tolerated drugs and rarely cause acute pancreatitis. Statin-induced pancreatitis is reported in medical literature as anecdotal case reports [7–10]. Pancreatitis that develops during treatment with the drug, resolves upon discontinuation of the drug, and the fact that it recurs upon readministration of the drug provides strong evidence that the drug causes pancreatitis [17]. Previously reported cases of statin-induced pancreatitis during the last decade are reported in Table 2.

Singh et al. [9] reported a case of possible rosuvastatin-induced pancreatitis and described it as a class effect of statins, as AP occurred during treatment with atorvastatin initially and later with rosuvastatin. Anagnostopoulos et al. [8] described a case of AP that occurred during pravastatin therapy, and AP recurred five months later when the patient resumed taking pravastatin of his own accord. Two case reports [7, 10] described the occurrence of AP in patients treated with combined salicylates and statins, which are commonly prescribed drugs for coronary artery disease.

Badalov and his colleagues [12] categorized the drugs that cause AP into 4 classes: class I drugs—at least 1 case report described a recurrence of AP with a rechallenge with the drug; class II drugs—there is a consistent latency in 75% or more of the reported cases; class III drugs—there were 2 or more case reports published but no rechallenge or consistent latency period; class IV drugs—these are similar to class III drugs, but only one case report was published. Based on this criterion, rosuvastatin should be considered a class I drug, as there is at least one case report of rechallenge with the drug. The Naranjo Adverse Drug Reaction Probability Scale [18] indicated a probable relationship between the occurrence of AP and use of rosuvastatin in this patient.

The exact mechanism of statin-induced AP is unknown, although a few case reports attribute it to drug interaction through CYP3A4 [19]. All statins except pravastatin are metabolized by CYP3A4. This could be one of the reasons that fewer cases of pravastatin-induced AP, as compared to AP induced by other statins, are reported in the medical literature [8]. Other potential mechanisms of statin-induced AP include rhabdomyolysis and myalgias [19]. AP can develop over a period of hours or years after the initiation of treatment with statins [20]. The period during which the onset of pancreatitis occurred varied considerably in previously reported cases of statin-induced AP. AP occurred during the first day of statin treatment in two case reports [4, 21], while in other cases, it occurred several years after the initiation of statin therapy. As noted in previous case reports, there was no consistent latency period for statin-associated acute pancreatitis. Several case reports of statin-associated pancreatitis suggested that statins must be stopped regardless of the latency period. The lack of a consistent latency period may even suggest possible toxic effects of drug metabolites on the pancreas [22]. This might be the possible mechanism in our patient who developed AP few days after starting rosuvastatin during first episode and eight weeks after restarting rosuvastatin during the second episode.

In conclusion, clinicians should strongly consider statin-induced pancreatitis, regardless of the duration of statin therapy, when other likely causes of AP are ruled out. Statins should be stopped and replaced with other drugs to prevent further episodes of AP. Statins should be cautiously used for the treatment of dyslipidemia in view of the potential side effects related to these drugs.

## Figures and Tables

**Table 1:** Laboratory data.

Serum chemistry	First admission	Second admission (8 weeks later)	Reference values
WBC (cells/cc)	**15,000 **	**11,400 **	4,800–10,800
Hemoglobin (gm/dL)	15.5	14.1	12–16
Glucose (mg/dL)	158	148	70–110
Potassium (mmol/L)	**3.1 **	4.0 mmol/L	3.5–5.1
Sodium (mmol/L)	137	138	135–145
Chloride (mmol/L)	101	104	100–110
Bicarbonate (mmol/L)	27	25	22–32
BUN (mg/dL)	14	15	8–23
Creatinine (mg/dL)	0.96	0.89	0.44–1.03
Calcium (mg/dL)	9.3	9.4	8.7–10.2
Amylase (U/L)	67	**168**	19–132
Lipase (U/L)	**82 **	**100**	8–56
Triglycerides (mg/dL)	58	64	<150
CEA (ng/mL)	N/A	1.3	<3

N/A: not Available.

**Table 2:** Previously reported cases of statin-induced pancreatitis.

Author	Patient age/gender	Associated drug/s	Drug rechallenge	Outcome
Belaiche et al. 2000 [4]	63/Male	Atorvastatin	No	Complete recovery
Tysk et al. 2002 [5]	36/Male	Fluvastatin	Yes: Recurrence	Complete recovery
McDonald et al. 2002 [6]	70/Male	Simvastatin & Fenofibrate	No	Fatal
Miltiadous et al. 2003 [7]	60/Male	Salicylate & Atorvastatin	No	Not available
Anagnostopoulos et al. 2003 [8]	56/Male	Pravastatin	Yes: Recurrence	Complete recovery
Singh et al. 2004 [9]	77/Female	Atorvastatin & Rosuvastatin	Yes: Recurrence with Rosuvastatin	Complete recovery
Antonopoulos et al. 2005 [10]	58/Male	Salicylate & Simvastatin	No	Complete recovery
Tsigrelis and Pitchumoni 2006 [11]	50/Female	Pravastatin	No	Complete recovery
Present case. 2012	67/Female	Rosuvastatin	Yes: Recurrence	Complete recovery
